# Address of Retiring President Harbaugh of Virginia State Veterinary Medical Association1Read at the annual meeting of the Virginia State Veterinary Medical Association, June 20, 1896.

**Published:** 1896-09

**Authors:** 


### ADDRESS OF RETIRING PRESIDENT HARBAUGH.[1]

In retiring from the presidency of this Association which I have held continuously since its incipiency, I must express my satisfaction on two particular points: the first is that I have earned a rest, and the second is that during my term of office we have accomplished all that we have undertaken for the advancement of our profession in this State.

To the set of officers who will to-day succeed the present incumbents we give a complete and smooth-working machine, which has been a power in this Commonwealth, and which should continue to be a power, increasing in strength and influence as numbers are added and as years roll by. It is almost incredible to think that this present Association with its present membership sprang from the handful of veterinarians who met in my office not quite two and a half years ago for the purpose of taking some step to check the incursion of the quacks with diplomas, and with the determination of doing what we professed to be able to do. Four of those six who organized this Association are still with us; they may be truthfully called the "old guard;" they are here to-day, and they have never failed to attend a meeting since we met to organize.

No matter where the meeting was held, whether up in the mountains in the extreme northern part of the State or down here by the seashore in the extreme east, these same earnest men have been on hand to contend for our personal rights and professional privileges. They recognize the fact that membership in this Association is not a mere name; they fully realize that this Association is a State institution, made such by the State legislature, and that membership in it is a responsible position; and now it is to be seen whether or not others have the courage to sacrifice time and money to carry on the work so well begun.

While it is true that pleasant social features had a prominent place at all our meetings, still we are not banded together for mere social pleasures. We were incorporated by the legislature to elevate the standard and advance the interest of our profession. We were made a State institution with a duty to perform

[1] Read at the annual meeting of the Virginia State Veterinary Medical Association, June 20, 1896.

to the people as well as to ourselves; and here the question may be asked: Have we performed that duty? In reply we can proudly point to the laws of our State, and say we have.

In a little more than two years we have accomplished much. Our calling has been lifted from its past degradation and put on an equality with the other learned professions; we are now recognized and legalized.

Questions in regard to contagious diseases are now referred to a member of the veterinary profession, instead of to the honest farmer, political granger, or a jury of laymen.

What we have done is done well, but our whole duty is not completed. We have a gigantic undertaking on our hands in our efforts to secure local dairy inspection for the different cities throughout the Commonwealth. We have to fight a monster which stretches forth its arms in all directions and clutches within its grasp all who can be controlled by fear, favor, or value received, and this monster is the wealthy breeding interest which makes a hobby of high-priced pedigree cattle until it tires of them and then unloads them on the unsuspecting dairyman to infect his smaller herd with tuberculosis.

Even from our standpoint there are two sides to this tuberculosis question. The first is the public health; and I care not whether a man believes there is much or little danger in using the milk or flesh of tubercular animals through risk of transmission of the disease to the human being, it is certain that such milk and flesh ought not to be used. Milk is part of the cow and therefore animal matter, and if the cow is tuberculous her milk is part of a diseased cow; it makes no difference how much it is boiled it is still part of a diseased cow, and should not be used for human food. The same proposition applies to meats from tuberculous animals, no matter how thoroughly sterilized, and it disgusts me to hear our would-be veterinary politicians talk of using such meats the same as they do for the lower classes in Europe, when we have meat to spare for the world. No, gentlemen, we are not in Europe and do not have to devour diseased products to prevent starvation. Let us be consistent and fight against all diseased animal products being used for human food. Another thing that surprises me is that there are veterinarians occupying high places who have the effrontery to tell us that milk from tuberculous herds, when fed to pigs, produces the same disease in them and that there is little danger of it producing the disease in human beings!

These are breeders' opinions, whether uttered by veterinarians, agricultural journals, or other hirelings. No man who sees the post-mortem lesions of a few tuberculous dairy cows wants milk from any such animals in his house, danger or no danger.

The other side of the question is the dairyman's side; we should protect him also while we are endeavoring to protect the public, and warn him against purchasing thoroughbred cows to increase the richness of his milk simply because he can buy the cow cheap. It would have been much better for all concerned if the price of the thoroughbred cow had remained high and beyond the reach of the average dairyman, for in that case there would have been an inestimably less amount of tuberculosis among our common cattle.

When I opened up this present campaign against tuberculosis I was well aware of the fact that I was sacrificing money, practice, and some friends; I knew that I was exposing myself to unjust criticisms, abuse, and falsehood; but it did not deter me; I have waged the war for more than two months, and the result is that the public is acquainted with facts they should have known long ago, and the great majority is on our side and is demanding what we are contending for, and, if I am not a false prophet, we will have it in the near future.

This question involves very hard work, but after what we have already accomplished nothing should be considered too hard to attempt, and if you go at it earnestly we will succeed. My plan has been to force the question in season and out of season, and make an object-lesson on every possible occasion.

When we have forced the institution of local dairy- and meat-inspection the breeders and dairymen will be only too glad to help us to force the State to make sufficient appropriation to begin an effort to stamp out the disease, or at least to check its spread, and when a sufficient number of States take up the question it will be discovered that the matter is a national one, and that the national government will have to take hold of it and handle it after the manner in which pleuro-pneumonia was handled, and the sooner such a step is taken the less it will cost the nation.

Mention should be made of the splendid work done by our efficient State veterinarian; many of you are aware of the stand he has taken in this war; he has put his shoulder to the wheel and has kept it there in spite of all threats and bluff of the opposition, and when our work is crowned with success Dr. Niles will come in for his full share of credit.

Now, gentlemen, I desire to warn you that we have stormy times ahead. Vigorous attempts will certainly be made to have our examining board law amended or repealed, and we must be on the lookout so as to be able to counteract every such attempt, even before it is made, if possible. We have already received notice that the law for the control and prevention of contagious diseases will be attacked, and we must never lose an opportunity to make friends for it, for you may rest assured that it makes an enemy of every man who suffers by it. We must look to the officers of the Association to guard its interests, and we will hold them responsible for any neglect.

